# Coming and going – Historical distributions of the European oyster *Ostrea edulis* Linnaeus, 1758 and the introduced slipper limpet *Crepidula fornicata* Linnaeus, 1758 in the North Sea

**DOI:** 10.1371/journal.pone.0224249

**Published:** 2019-10-24

**Authors:** Sarah Hayer, Andreas Bick, Angelika Brandt, Christine Ewers-Saucedo, Dieter Fiege, Susanne Füting, Ben Krause-Kyora, Peter Michalik, Götz-Bodo Reinicke, Dirk Brandis

**Affiliations:** 1 Zoological Museum, Christian-Albrechts-University, Kiel, Schleswig-Holstein, Germany; 2 Zoological Collections of the University Rostock, Institut for Biosciences, General and Systematic Zoology, Rostock, Mecklenburg-Vorpommern, Germany; 3 Senckenberg Research Institute and Natural History Museum, Frankfurt am Main, Hessen, Germany; 4 Evolution and Diversity, Institut for Ecology, Goethe-University of Frankfurt, Frankfurt am Main, Hessen, Germany; 5 Museum for Nature and Environment, Lübeck, Schleswig-Holstein, Germany; 6 Institut of Clinical Molecular Biology (IKMB), Christian-Albrechts-University, Kiel, Schleswig-Holstein, Germany; 7 Zoological Museum of the University Greifswald, Greifswald, Mecklenburg-Vorpommern, Germany; 8 German Oceanographic Museum, Stralsund, Mecklenburg-Vorpommern, Germany; State Museum of Natural History, GERMANY

## Abstract

Natural history collections are fundamental for biodiversity research as well as for any applied environment-related research. These collections can be seen as archives of earth´s life providing the basis to address highly relevant scientific questions such as how biodiversity changes in certain environments, either through evolutionary processes in a geological timescale, or by man-made transformation of habitats throughout the last decades and/or centuries. A prominent example is the decline of the European flat oyster *Ostrea edulis* Linneaus, 1758 in the North Sea and the concomitant invasion of the common limpet slipper *Crepidula fornicata*, which has been implicated to have negative effects on *O*. *edulis*. We used collections to analyse population changes in both species in the North Sea. In order to reconstruct the change in distribution and diversity over the past 200 years, we combined the temporal and spatial information recorded with the collected specimens contained in several European natural history collections. Our data recover the decline of *O*. *edulis* in the North Sea from the 19^th^ century to the present and the process of invasion of *C*. *fornicata*. Importantly, the decline of *O*. *edulis* was nearly completed before *C*. *fornicata* appeared in the North Sea, suggesting that the latter had nothing to do with the local extinction of *O*. *edulis* in the North Sea.

## Introduction

*Ostrea edulis* Linnaeus, 1758 or the European flat oyster has been used as food source by humans for centuries. Shells have been found during excavations of Mesolithic kitchen middens and in settlement remains of Vikings as well as Romans [1–5]. In the German North Sea, oyster fishery was one of the first recorded commercial fisheries in the 12^th^ and 13^th^ century [5–6]. In later centuries, the demand for oysters remained high and oyster fishery along the North Sea coasts continued to increase and become more efficient [5]. However, due to the constant fishing of adult oysters, the spat fall declined over the years [5]. In 1868, the Prussian King and German Emperor Wilhelm I. decided to make the attempt to breed oysters commercially. Karl August Möbius, professor of Zoology and director of the Zoological museum in Kiel (Germany) at the time, received a research contract to investigate the possibility of artificial oyster farming in the German North Sea. Additionally, he was asked to find a way to improve the productivity of the native oyster banks due to the increased demand for oysters. Therefore, he visited oyster beds in the North Sea on the coasts of Germany, France and England to fulfil his contract and brought many specimens of *O*. *edulis* back to Kiel and other museums. He concluded that oyster farming as it was carried out in France and Great Britain was not possible at the German coast, and oyster production in the North Sea was already maximized [7]. In 1882, oyster fishery had to be stopped on the German island Sylt, because the oyster beds were overaged [8]. Despite measures to protect the oyster population from overexploitation, oyster fishery continued to decline dramatically. Most natural populations of the European oyster went extinct in the North Sea in the 1940s [5,9].

Until the beginning of the 20^th^ century, *O*. *edulis* was commonly found in the shallow regions of the Atlantic coasts from Norway to North East Africa as well as from the Mediterranean Sea to the Black Sea [2,10–12]. Nowadays, *O*. *edulis* is found in large numbers in the Limfjord, which is the only surviving natural population in the North Sea, but mostly in commercial oyster farms on the European Atlantic coast or in very small and endangered natural populations in, for example, Norway and Sweden [5]. Recently, there are observations of *O*. *edulis* in Danish offshore wind farms, hinting at a possible re-colonization of the North Sea [2,5,13–15].

It remains a mystery why the European flat oyster has not returned to the North Sea sooner, since the environmental conditions for a successful establishment have not changed. *O*. *edulis* generally inhabits the intertidal zone to a depth of 20 metres, but has been found at depths up to 50 metres [5,12]. The individuals often occurred in large beds on muddy-sand, muddy-gravel and firm grounds, where they feed on plankton [7,12]. *O*. *edulis* normally needs salinities above 30 ‰, but for short periods of time, it tolerates salinities between 16 ‰ and 19 ‰ in estuaries [5,16]. The European flat oyster is a protandric hermaphrodite, generally changing sex once a year in the North Sea [5,12]. An *O*. *edulis* individual may begin the new season either as a male or as a female [2]. Some *O*. *edulis* function as males early in the spawning season and change later to females before becoming males again in the next season. Female individuals produce up to 1 million eggs per spawning and release them into the inhalant chamber, where they are fertilised by the indrawn sperm of neighbouring male individuals [2]. Following an incubation period of 8–10 days, depending on water temperature, larvae are released into the environment and spend 8 to 10 days as pelagic dispersal stages before they settle down on a suitable substrate. Appropriate larval growth and survival rates are obtained in 20‰ salinity and a minimum temperature 15°C—16°C, although they can survive at salinities as low as 15‰ [5,12].

Numerous reasons for the extinction of natural oyster beds are hypothesized. The overexploitation is the most favoured reason, but strong winters, diseases and invasive species as competitors have been speculated to play a role as well [5,7,9,15,17]. One invasive species that was thought to threat the oyster populations was *Crepidula fornicata* Linnaeus, 1758, the common limpet slipper [9]. This snail is commonly found in the intertidal zones and is also a filter feeder, hence was feared to be a feeding competitor for *O*. *edulis* [9,18–25]. It was introduced to Europe together with the Pacific oyster, *Magallana gigas* (Thunberg, 1793), that was imported for the first time in 1870 to revive the European oyster fishery [9,24]. *C*. *fornicata* was first discovered on the German coast in 1934 [9,19,24,26]. While it was also introduced to other regions of the world, it spread rapidly in Europe, where it is established now and occurs from South Norway to Spain [9].

To reconstruct the demographic distribution and abundance of both *O*. *edulis* and *C*. *fornicata* in the past, we surveyed museum collections across Europe. The historical collections of museums are the basis of taxonomic and biogeographic research as well as applied environmental research [27–29]. They are a valuable heritage in their historical, biological and cultural references. The collections document the dynamics of change of the biosphere. They preserve evidence of changes in biodiversity, either through evolutionary processes in geologically long or short periods, or through man-made transformation of habitats [27]. Thus, the collections have, among other things, a function as ecological archives by documenting ecological condition in a particular place and time.

The aim of our study is to investigate a possible connection between the extinction of *O*. *edulis* in the North Sea and the arrival of the invasive common limpet slipper *C*. *fornicata*. To this end, we reconstruct the historical distribution of *O*. *edulis* in the North Sea from the 19^th^ century to the present based on speciments of museum collections.

## Material and methods

### Data preparation

We used approximately 1750 individual clamshells of *Ostrea edulis* and 739 individual shells of *Crepidula fornicata* collected between the 1820s and 2018. Table 1 lists the number of collected specimens from every museum used in this study, where they are permanently reposited. The details of the location of every specimen are given in the S1–S5 Tables (supporting information). Records for both species were reviewed and the identification was verified. In order to reconstruct all relevant specimen data, we further surveyed the archives of the respective museums.

**Table 1:** Numbers of collected specimens and collection records of *Ostrea edulis* and *Crepidula fornicata* from cooperating museums and from public databases of the museums in London (GB), Leiden (Netherlands) and Paris (France).

Museum/collections	Museum acronym	Records of *O*. *edulis*	Indiv. of *O*. *edulis*	Records of *C*. *fornicata*	Indiv. of *C*. *fornicata*
Senckenberg Natural History Collection, Dresden, Germany	SNSD	1	1	8	20
Senckenberg Natural History Museum, Frankfurt, Germany	SMF	2	175	5	8
Zoological Museum Greifswald, Germany	ZIMG	2	> 3	/	/
Centre of Natural History, Hamburg, Germany	ZMH	20	68	/	/
Zoological Museum, Kiel, Germany	ZMK	93	509	11	107
Naturalis Biodiversity Center, Leiden, Netherlands	NMNL	146	851	97	> 495
Natural History Museum, London, UK	NHML	3	6	10	70
Museum for Nature and Environment, Lübeck, Germany	MNUL	3	18	1	5
Zoological Collections of the University Rostock, Germany	ZSRO	14	> 85	/	/
German Oceanographic Museum, Stralsund, Germany	DMM	6	28	13	> 34
Muséum National d’Histoire Naturelle, Paris, France	MNHN	5	5	/	/

In order to determine whether *O*. *edulis* was collected alive or dead, the shells were consulted unless this information was given on the original labels. If the right and left valve of the shell or musculature tissue were present or the periostracum was intact, the shells were labelled as being found alive. On the contrary, if shells were single, overgrown on the inner surface of the shell by epifauna, damaged or heavily infested by parasites the shells were labelled as being found dead. These findings are referred to as 'empty shells' later on. If neither of these circumstances occurred or the material could not be checked, the status was considered to be unknown.

To determine species distribution, the original geographic coordinates were used when provided. When no geographic coordinates were given, google maps was used to infer the geographical coordinates from the location description. In some cases, the descriptions of localities were too ambiguous, hence two values for each longitude and latitude were calculated: The most possible northern and southern for latitude and the eastern and western most possible location for longitude.

### Plotting the data on maps

To reconstruct the historical distribution of the species in the North Sea, only the most northern and western coordinates for each specimen were used. The maps were constructed in R Studio version 1.1.453 [30] and the additional package ‘ggplot2’ was used for graphical output [31]. The graphics were edited using Affinity Designer (version 1.6.1, Serif, Nottingham, England).

### Graphical output and statistics

All analyses were conducted in R Studio version 1.1.453 [30] using the ‘ggplot2’ package for graphical output [31]. To investigate the relationship between the collection records of live *O*. *edulis* and *C*. *fornicata* over time, we calculated the number of records per decade and plotted the results on a linear plot using the ‘ggplot2’ package. To analyse if the annual number of shells collected declined over time, we modelled a linear regression with the ‘lm’ function of the ‘stats’ package between the collection years and the number of shells collected. Therefore, we had to exclude the collection records without information on numbers of shells. We also calculated the relationship between the collection year and whether *O*. *edulis* was found dead or alive using a logistic regression. We used the ‘glm’ function of the ‘stats’ package to model a logistic function. In order to minimize bias, we calculated both models with the whole dataset and without the oysters collected by Möbius, since he collected consistently over 17 years.

## Results

**Fig 1:**
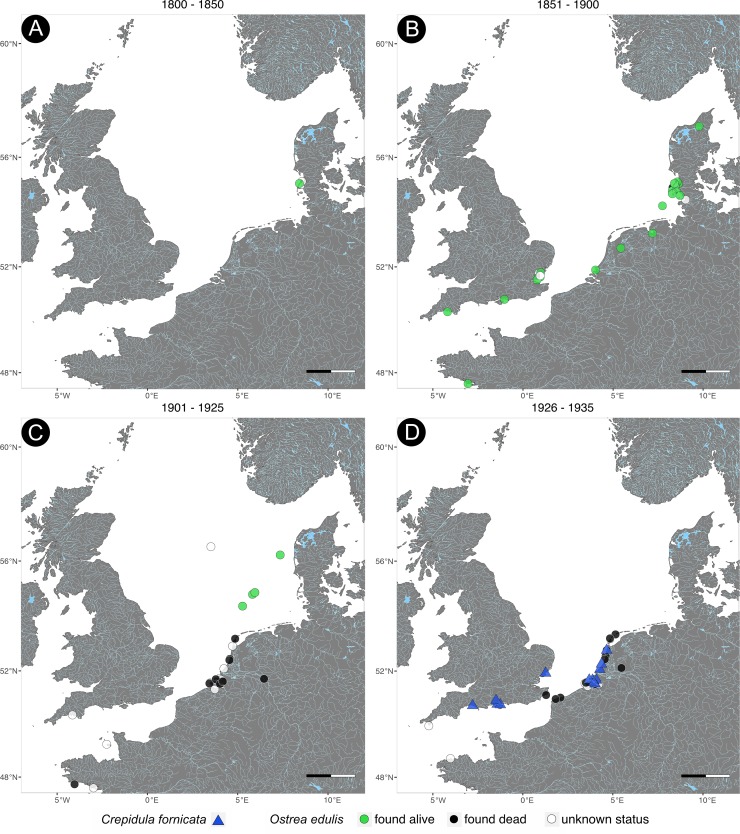
Historical distribution of *Ostrea edulis* and *Crepidula fornicata* from the 1820s to 1935. Time series of the distribution of both species in the North Sea. *O*. *edulis* was mapped according to its sampling status. Scale bar = 100km.

**Fig 2:**
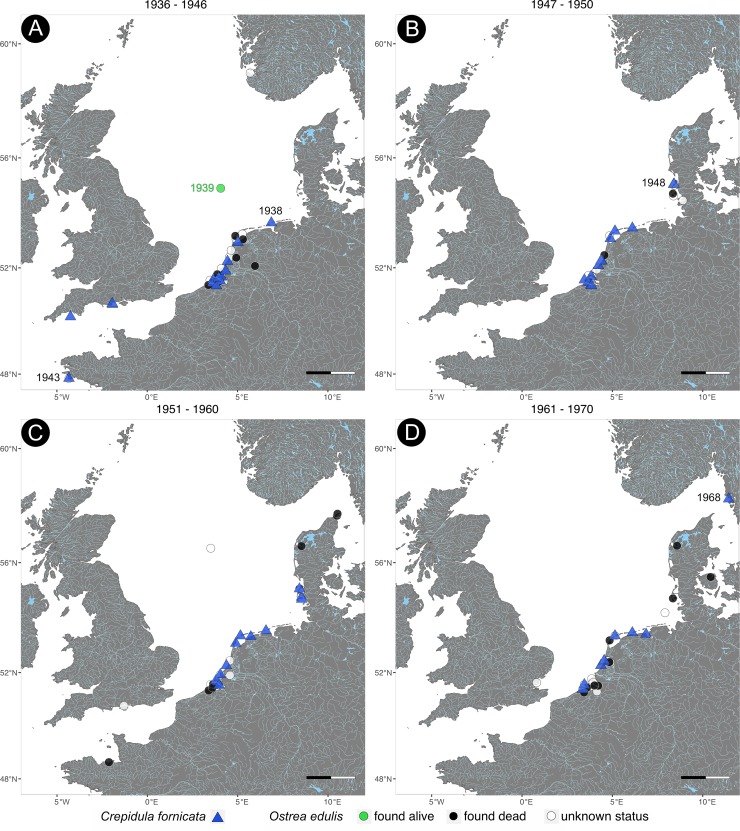
Historical distribution of *Ostrea edulis* and *Crepidula fornicata* from 1936 to 1970. Time series of the distribution of both species in the North Sea. *O*. *edulis* was mapped according to its sampling status. Scale bar = 100km.

**Fig 3:**
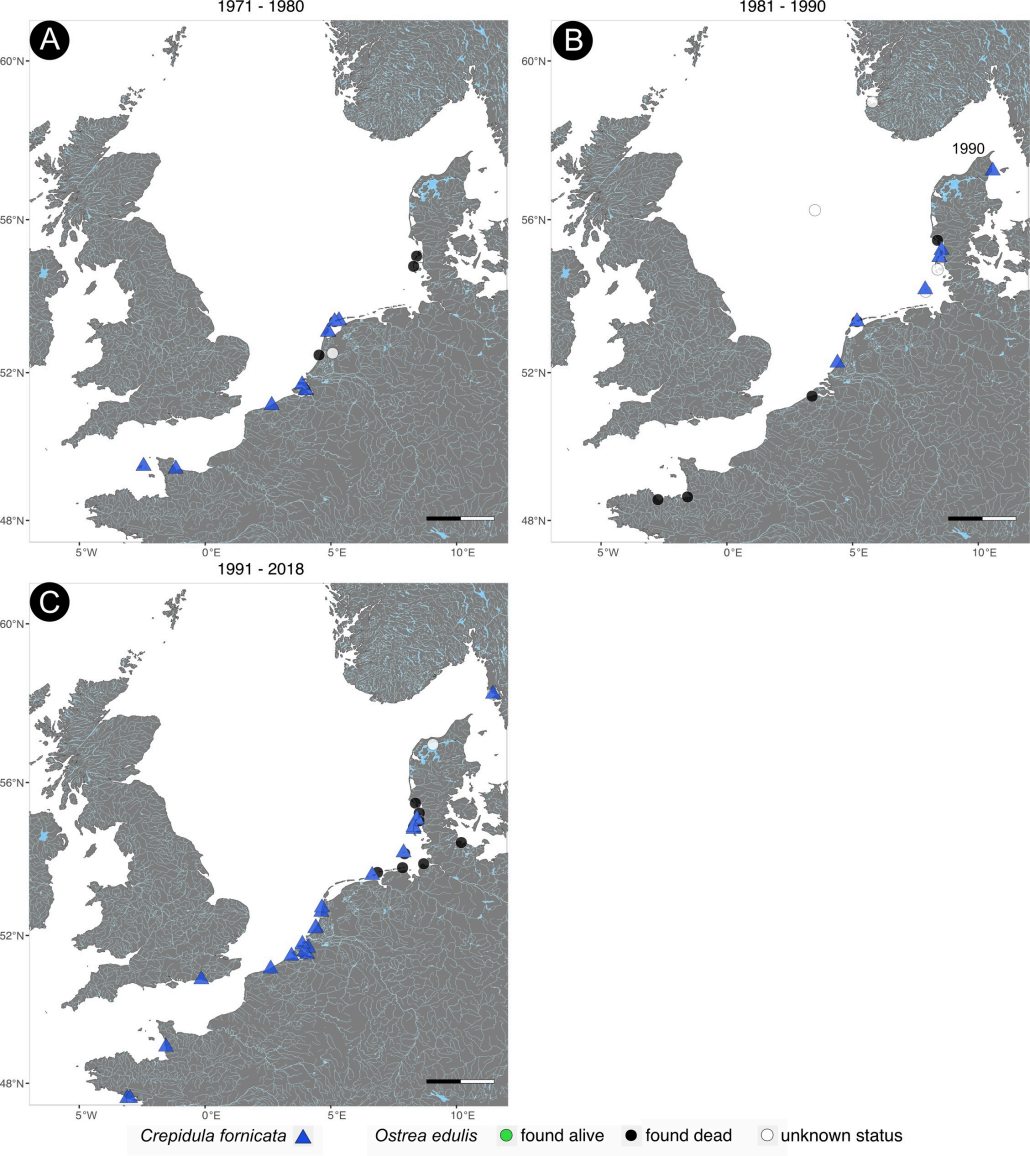
Historical distribution of *Ostrea edulis* and *Crepidula fornicata* from 1871 to 2018. Time series of the distribution of both species in the North Sea. *O*. *edulis* was mapped according to its sampling status. Scale bar = 100km.

The investigated museum collections contain specimens of *O*. *edulis* from 19^th^ to the 21^st^ and *C*. *fornicata* from the 20^th^ to 21^st^ century with a wide distributional range across Europe (Table 1; see S1 Fig). In the following, we will focus on the historical distribution of *O*. *edulis* found alive and *C*. *fornicata* in the North Sea in the 19^th^, 20^th^ and 21^st^ century (see Figs 1–3).

The earliest collected specimen of *O*. *edulis* available dates back to the 1820s and was known to be six years old when collected in the Northfrisian Wadden Sea (Fig 1A). Between the years 1868 and 1885 the full North Sea area was sampled and *O*. *edulis* was collected alive from the coasts of Denmark, Germany, the Netherlands, France and England (Fig 1B). For instance, the ‘true native’ market oyster was bred in the estuaries of the river Roach (Essex, England) and river Colne, a tributary of the Thames. Another market oyster called ‘the Nore’ that had already gone extinct at that time, was collected from the Thames estuary in 1869. An oyster labelled to be very old was collected from the Herne Bay (Kent, England), although it originated from the English Channel and was put there to refresh the oyster beds in Herne Bay (Fig 1B).

Between 1868 and 1886, *O*. *edulis* was commonly found in the German ‘North Frisian Wadden Sea’ and at the West Coast of Schleswig-Holstein (Germany), where several native oyster beds were known and documented [5, 17] (see Fig 1B). Some of those individuals were collected from the age of 14 days after metamorphosis to 30 years in short intervals. As an example, oysters of the age of one to two years were collected on stones on the oyster bed ‘Morsum Odde’ after a very mild winter. There, they were flooded 2,5 metres at high tide and lied on dry ground on low tide. Others were collected with parasites, for example, 15 individuals were infested by boring sponges and had barnacles attached to their shells when they were collected at the oyster beds of Sylt in August 1876. *O*. *edulis* was sampled in the Oosterschelde labelled as market oysters in March 1878 (Fig 1B).

In 1903 and 1905, *O*. *edulis* were sampled in the North Sea by the ‘commission of the scientific investigation of the German seas’ on behalf of the former German royal ministry of agriculture (Fig 1C). Those oysters were dredged alive in depths of about 30 to 78 metres by the research vessel ‘Poseidon’. After 1905 the amount of live oysters decreased and empty shells were found at the coasts of the Netherlands, Germany, Lebanon, Denmark, Italy, France, Croatia and Greece (Figs 1–3). Few live oysters were collected in the North Sea part of ‘Oestergronden’ in 1939 (Fig 2A). Cultivated individuals of *O*. *edulis* were bought from the oyster farm in Galway (Ireland) in 2017 (see S1 Fig).

**Fig 4:**
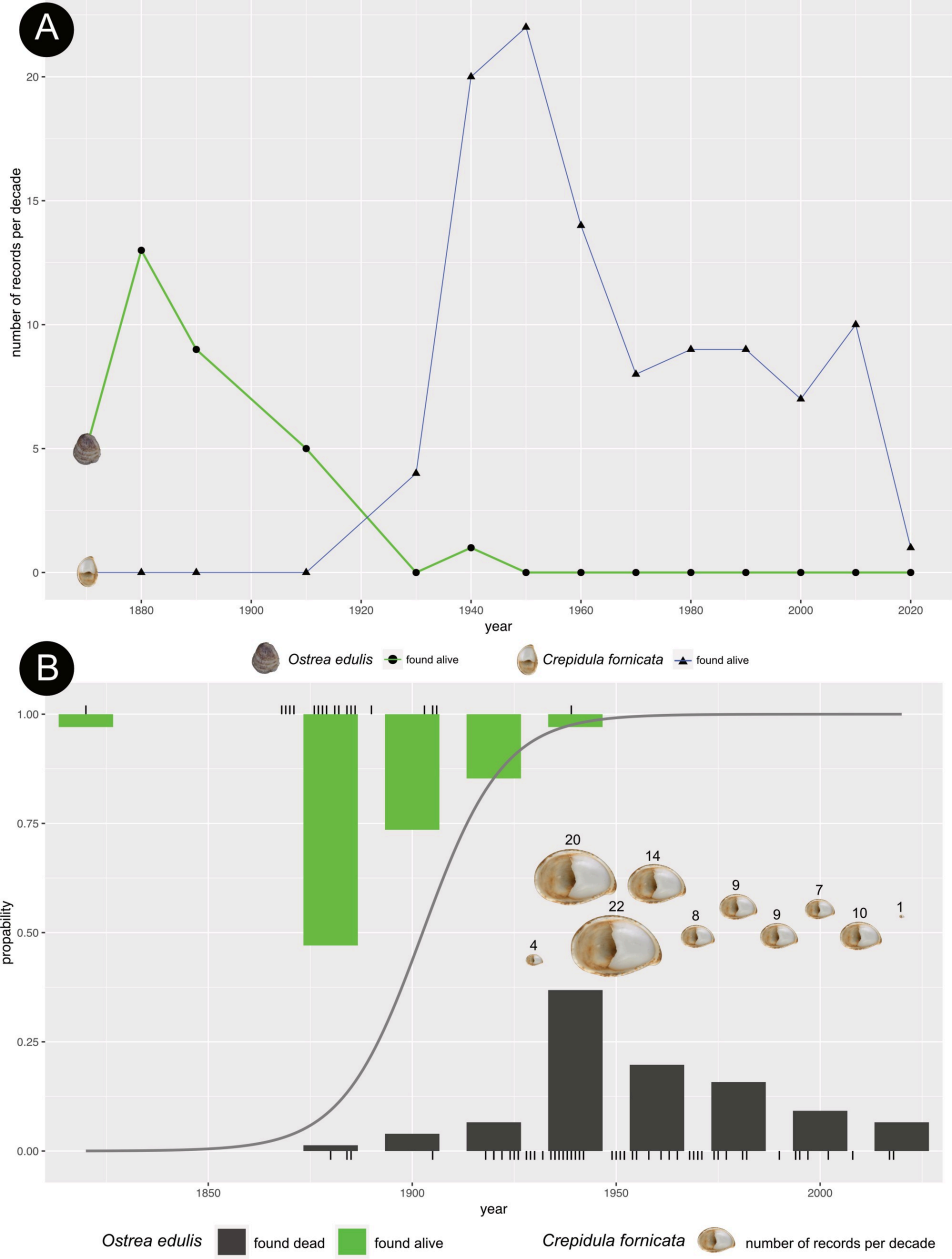
Graphical output of *Ostrea edulis* and *Crepidula fornicata*. (A) Line plot of the collection records per decade of live *O*. *edulis* and *C*. *fornicata* from the North Sea housed in natural history collections over time. The values on the y-axis display the number of records collected each decade. The values on the x-axis display the year of sampling. (B) Logistic regression plot of the collection records of *O*. *edulis* in the North Sea over time. Additionally, the number of museum records of *Crepidula fornicata* per decade is displayed. The values on the y-axis display the conditional density of oysters found alive (= 1) or dead (= 0). The values on the x-axis display the year of sampling. A logistic regression line shows the decreasing probability of oysters found alive over the years.

The results show that the number of museum records of live oysters had already declined dramatically when the records of *C*. *fornicata* increased (Fig 4A). According to the results of the logistic regression model, the number of collection records of *O*. *edulis* per year declined significantly over time (p-value < 0.05, Table 2, see Fig 4B). The number of museum records per decade of *C*. *fornicata* in the North Sea is represented in the logistic regression plot by the size of *C*. *fornicata* shells (Fig 4B). These results remain significant when the collections of Möbius were removed from the dataset (see Table 2). However, the linear regression model reveals that the number of individual shells of *O*. *edulis* collected alive did not decrease over the years (p-value > 0.05, see S2 Fig, S6 Table). This result is comprehendible as the number of individual oyster shells added to a museum collection were sampled in random numbers since they were collected by different collectors but a collection record of an oyster shell represents always a finding of the animal at a place.

**Table 2:** Detailed results of the logistic regressions from the dataset of *Ostrea edulis*.

	Estimated coefficient	Standard error	t-value	p-value
Complete data base				
intercept	-191.91907	35.65546	-5.383	7.34e-08 ***
Year	0.10088	0.01874	5.384	7.28e-08 ***
Without Möbius oysters (1868–1885)				
Intercept	-320.74965	99.86855	-3.212	0.00132 **
Year	0.16788	0.05212	3.221	0.00128 **

Regressions were calculated with the complete dataset and without the oysters collected by Möbius. Provided are estimated coefficients, standard errors, t-values and p-values for collection years as a function of the number of shells collected.

Note: high significance = ***; firm significance = **

*C*. *fornicata* was first recorded at Camperduin-Petten (the Netherlands) on 5^th^ September 1926 and in Davercourt (Essex, England) on 14^th^ September 1929 (Fig 1D). From there, it spread along the coasts of England and the Netherlands until *C*. *fornicata* was found at the French Atlantic coast and at the North Sea border to Germany in the late 1930s (Figs 1D and 2A). In August 1948, it was collected at Königshafen on the German island Sylt providing evidence for the first individual on Sylt (Fig 2B). *C*. *fornicata* spread again further north and was first documented at the Gullmarnfjord (Sweden) on 11^th^ October 1968 with a salinity of 32 (Fig 2D). On 10^th^ September 1990, the slipper limpet made its way into the Kattegat and was found at the beach of Lyngså (Denmark, see Fig 3B). Today it can be found along all coasts of the North Sea from France to Sweden (Fig 3C).

## Discussion

Our study shows a significant decrease of live individuals of *O*. *edulis*, a significant increase of empty oyster shells as well as individuals of *C*. *fornicata* in the North Sea at the beginning of the 20^th^ century (Fig 4A and 4B). Importantly, we can show that *C*. *fornicata* appeared after the breakdown of the *O*. *edulis* population in the North Sea. The number of museum specimens of *C*. *fornicata* increased in the 1920s (Fig 4A). At that time, the number of collection oysters had already declined dramatically (Fig 4A).

At the beginning of the 19^th^ century, few *O*. *edulis* specimens were collected by museums (Figs 1A and 4B). This contradicts the literature stating that the European flat oyster was commonly found in the North Sea [5,7]. For example, oysters were fished with estimated annual catch rates of over 20 million in Great Britain at the end of the 18^th^ century [2,32]. One explanation for this discrepancy between literature and our results could be the fact that few *O*. *edulis* specimens were collected and catalogizied in museum collections in the early 19^th^ century. Native and common species are often underrepresented in museum collections because of selective sampling [33]. In most cases only rare and outstanding species were collected to document the change in ecosystems [33]. Since a change in the abundance of *O*. *edulis* was not expected due to the belief in inexhaustable oyster beds, this species was uninteresting for museum collections. In addition, older collections and their documentation are often lost. This is an inherent bias of museum collections that cannot be avoided, only recognized.

By the middle of the 19^th^ century, an abundance of live *O*. *edulis* was collected, but no empty shells are recorded (Fig 1B). These records trace back to Karl August Möbius, professor of Zoology and director of the Zoological museum in Kiel (Germany) at the time. He only collected live oysters, because he was interested in the oysters' habitat requirements. This is also an example of biased sampling, because K. A. Möbius was collecting with a clearly defined question of improving oyster farming in the German North Sea. In 1880, our results show that the number of collected oysters is with 13 records per decade at its highest (Fig 4A and 4B). Towards 1900, the numbers of live oysters are declining rapidly (Fig 4A and 4B). This result is reflected by the literature stating that 4–5 million oysters were fished on the German island of Sylt in the middle of the 19^th^ century, before fishing had to be stopped in 1882 because of low catch rates [8]. In Germany, the decline of *O*. *edulis* became especially apparent when other oyster beds on the East Frisian Islands were overfished by 1855 and fishing was no longer possible as well [7].

At the beginning of the 20^th^ century, no live oysters were found in the shallow water oyster beds in the North Sea (Fig 1C). The only specimens of *O*. *edulis* found alive were collected by the ‘Commission of the Scientific Investigation of the German Seas’ from depth of 30 to 78 in the North Sea between the German island Helgoland and the Danish sand bank Horns Rev. These individuals were partly covered by barnacles and young oyster spat indicating to belong to healthy oyster beds. According to literature, these offshore oyster beds were discovered in the middle of the 19^th^ century and spread out over 21 000 km^2^ [5,7,34,35]. Soon after the discovery of the new and profitable oyster beds, commercial fishery exploited them within a century [17]. Hagmeier and Kändler ([17]: 70) state: "During the cruise with the research vessel 'Poseidon' in March observations were made of the oysterbeds 'Austerngrunde' in the North Sea. Dredging along half a sea mile was completely unsuccessful, hence several attempts have been made resulting in 42 oysters in total (…)".

In the 1930s, our results recorded only one record of live oysters, but the largest number of empty shells of *O*. *edulis* was documented at that time (Figs 1D and 4B). At the same time, the number of *C*. *fornicata* increased rapidly up to 22 records per decade (Figs 1D, 4A and 4B). Originally, *C*. *fornicata* first established itself in Ireland and England in 1870, but the museum collections recorded the first individual 60 years later [24]. This pattern of rapid spread and a subsequently stable level is typical for neozoan species that adapt to a new environment and can be observed in many invasive species [36–38]. Many neozoa pass unnoticed during the first stage of invasion, because the populations are small and localized after introduction (‘lag-phase’). This may be an explanation why the museum collections have no records of *C*. *fornicata* before 1926, although the introduction date is known to be 1870 [24]. Another explanation could be a lack of data in this study because not all European museums were included. For this study, only few large museums containing North Sea material were included that already digitalized their collections and made them public. Most museums only started recently to digitalize their collections and thus possible records of *C*. *fornicata* could have been documented but are not included in this data set.

The second stage of invasion, which often rises public awareness, is the expansion stage [38–41]. During this stage, the population is growing rapidly, which is also documented by the museum collections of *C*. *fornicata*. The subsequent persistence stage is distinguished by natural fluctuations of the population size, which can partly also be observed in *C*. *fornicata* (Fig 4A). Here, the numbers decline after the rapid increase, which could also be an artefact of collection events. As Guralnick and Van Cleve [33] state, only outstanding or invasive species were collected to document the change in ecosystems. After the first 30 years of collection events, the excitement about the spread of the invasive *C*. *fornicata* may have passed and collection events declined thus the declining number of collection records. This assumption is supported by our results showing only one individual recorded for the last decade, although *C*. *fornicata* is commonly found on the North Sea coasts nowadays [37,42].

The arrival of *C*. *fornicata* as an additional competitor could have been another burden for *O*. *edulis*, which could have accelerated the decline of the populations of *O*. *edulis*. The fishing industry feared *C*. *fornicata* to be harmful for the oyster beds, especially because they occurred in huge numbers soon after arrival (see Fig 4B) [9]. Since the snail is a suspension feeder filtering phytoplankton and particulate organic matter as is the European flat oyster, it was assumed that *C*. *fornicata* could act as a feeding competitor, but this could not be verified [9,24,43,44]. It has also been shown that adult individuals of *C*. *fornicata* are able to ingest large particles such as *O*. *edulis* larvae, however, since *O*. *edulis* is also feeding on planktonic larvae and thus also on *C*. *fornicata* larvae, the predation effect is levelled out [7,12,45]. Another assumed risk represented by *C*. *fornicata* for oyster beds was that it would change the environment by massive production of pseudofaeces, enriching the soft sediments in oyster beds [9,46].

On the contrary, it would also be possible that the dying oysters gave room for the common limpet slipper to spread, because the oysters were strong competitors. This hypothesis would be supported by our results that illustrate that the population of *O*. *edulis* was already endangered when the numbers of *C*. *fornicata* are increasing rapidly (Fig 4A).

After most oyster beds went extinct in the 1940s, only one population of *O*. *edulis* survived in the Limfjord (Denmark) [5]. The results recorded some individuals found alive in the Limfjord in December 1869, but mostly empty shells were washed ashore in the middle of the 20^th^ century. Literature states that the population of *O*. *edulis* in the Limfjord started to grow after a storm in 1825 connected the previously isolated Limfjord with the North Sea [5]. After a few decades the population was big enough for commercial fishing. Despite being fished the population is still thriving nowadays [5].

Thus, we cannot confirm that the European flat oyster became extinct in the North Sea in the 1940s. Individual specimens can still be found in the North Sea [2,5]. This is also supported by recent observations in off shore wind parks in Horns Rev (Denmark) and near Egmond aan Zee in the Netherlands [47,48]. They were established in 2002 and 2006 respectively. In both cases, *O*. *edulis* was found in the intertidal zone on the monopiles of the offshore wind parks. The museum collections documented the presence of the oyster in the past in both areas, so it can be concluded that oyster spat from remaining oyster beds settled down on this new habitat. Because of these new findings, we support the results of habitat controls that conclude that *O*. *edulis* is found very rarely and is threatened by extinction rather than being extinct [49,50].

The results of this study show the importance and scientific potential of museum collections. Combining thorough collection documentation and existing public collection databases provided a unique basis to reconstruct the historical distribution of *O*. *edulis* and *C*. *fornicata*. With this dataset, the spread of the invasive species *C*. *fornicata* and the decline of the native species *O*. *edulis* could be reconstructed geographically from the 1820s to the year 2018. For the future, it would be helpful to have a public database combining the museum collections for further studies about *O*. *edulis*, *C*. *fornicata* or future studies about other North Sea species, since those data bases provide valuable historical facts.

## Conclusion

Our study reveals the value of natural history collections. By combining public records of Natural History museum all over Europe and records from the collections of small museums in Northern Germany (NORe e.V museums), we were able to reconstruct the historical distribution of *O*. *edulis* in the North Sea from the 1820s to the year 2018. Furthermore, we could reconstruct the process of invasion of *C*. *fornicata* in Northern Europe, which does not take place until *O*. *edulis* records of live shells had already declined dramatically and the records for dead shells increased. Our data suggest that the common limpet slipper is not responsible for the near extinction of *O*. *edulis* in the North Sea, since the numbers of *C*. *fornicata* exploded not until the 1940s –ten years after the local extinction of *O*. *edulis* in the North Sea.

## Supporting information

S1 Fig Overview of the distribution of *Ostrea edulis* and *Crepidula fornicata* in Europe based on data of different museum collections across Europe. (A) historical distribution of *O*. *edulis* on one map between the 1820s and 2018 coloured by the museum collection; (B) historical distribution of *C*. *fornicata* on one map between 1926 and 2017 coloured by the museum collection; scale bar = 500km.
(TIFF)

S2 Fig Scatterplot of the number of shells of *O*. *edulis* from the North Sea housed in natural history collections over time. The values on the y-axis display the number of shells collected annually on a logarithmic scale. The values on the x-axis display the year of sampling.
(TIFF)

S1 Table Details of *Ostrea edulis* of the 19^th^ century, chronologically ordered. (XLSX)

S2 Table Details of *Ostrea edulis* of the 20^th^ century, chronologically ordered. (XLSX)

S3 Table Details of *Ostrea edulis* of the 21^st^ century, chronologically ordered. (XLSX)

S4 Table Details of *Crepidula fornicata* of the 20^th^ century, chronologically ordered. (XLSX)

S5 Table Details of *Crepidula fornicata* of the 21^st^ century, chronologically ordered. (XLSX)

S6 Table Detailed results of the linear regression tests from the data set of *Ostrea edulis*. The test was calculated with the complete data set and with the oysters collected by Möbius removed. Provided are estimated coefficients, standard errors, t-values and p-values for collection years as a function of the number of shells collected. Note: high significance = ***; low significance = *; no significance = no asterisks.
(DOCX)
