# Supplementary figures and images for: Coming and going – Historical distributions of the European oyster Ostrea edulis Linnaeus, 1758 and the introduced slipper limpet Crepidula fornicata Linnaeus, 1758 in the North Sea
Source: PLoS One. 2019 Oct 24;14(10):e0224249. doi: 10.1371/journal.pone.0224249 (PMC6812771; doi:10.1371/journal.pone.0224249)

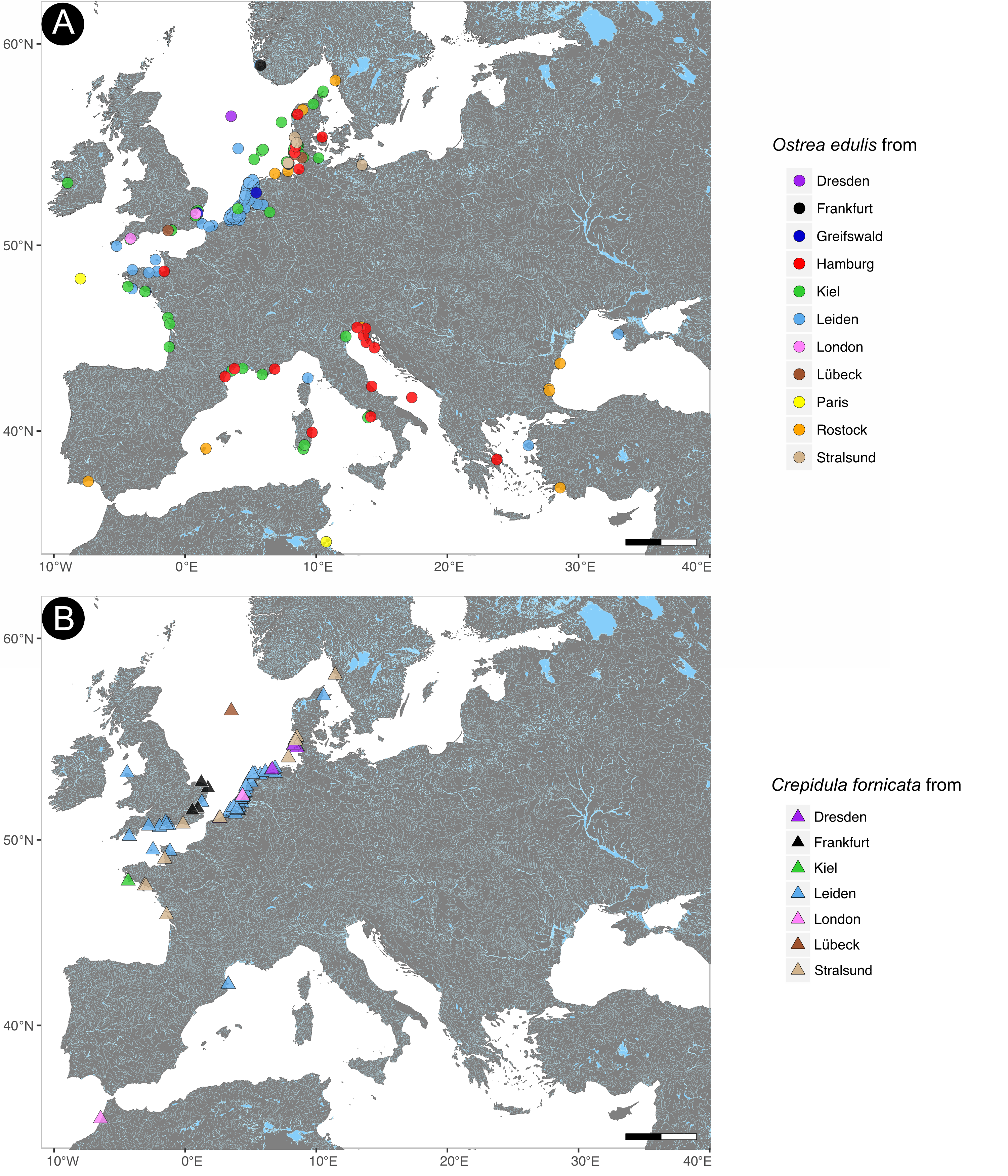

Supplement: S1 Fig — (A) historical distribution of O. edulis on one map between the 1820s and 2018 coloured by the museum collection; (B) historical distribution of C. fornicata on one map between 1926 and 2017 coloured by the museum collection; scale bar = 500km. (TIFF) [file pone.0224249.s001.tiff]

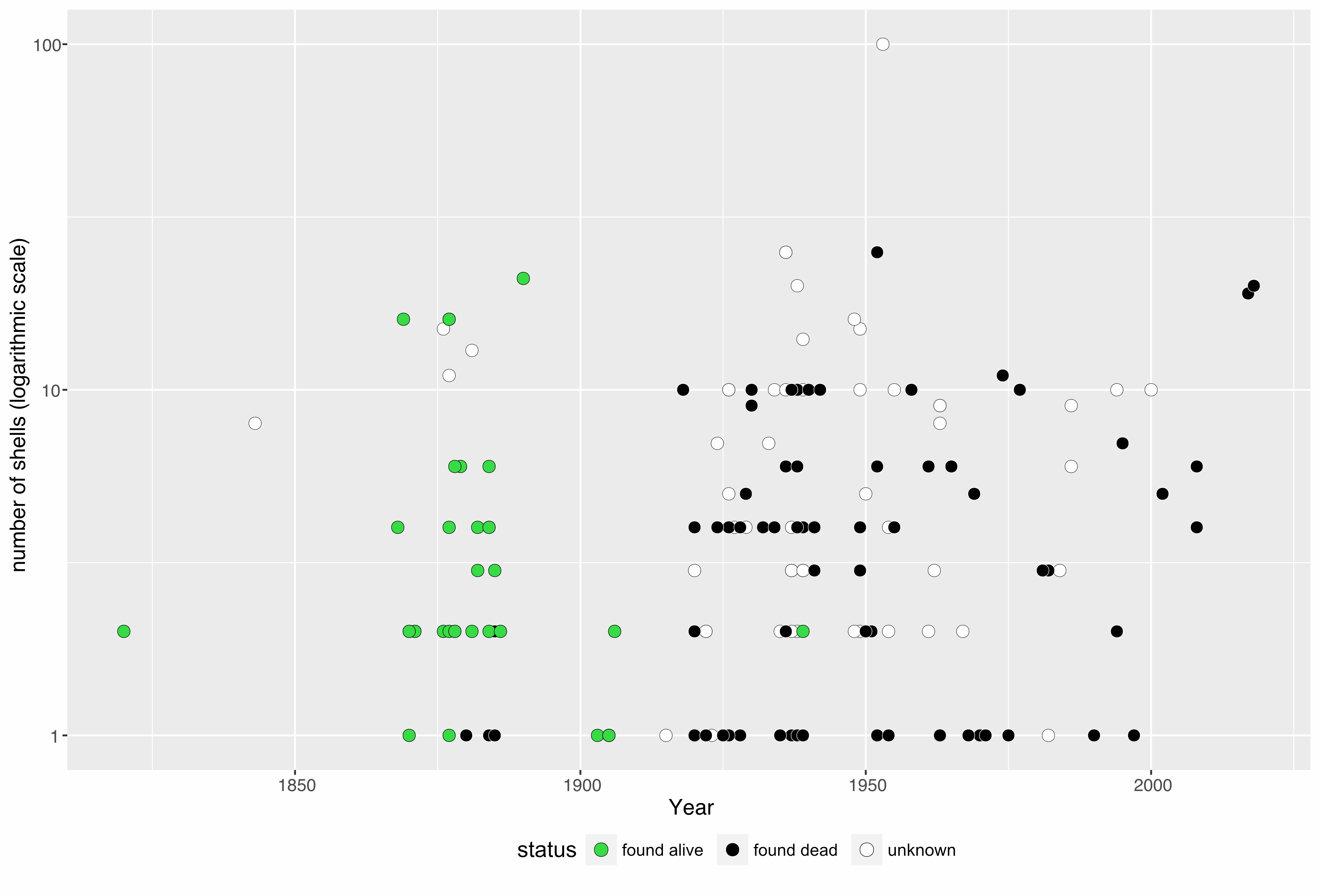

Supplement: S2 Fig — The values on the y-axis display the number of shells collected annually on a logarithmic scale. The values on the x-axis display the year of sampling. (TIFF) [file pone.0224249.s002.tiff]
