# Supplementary material for: Coming and going – Historical distributions of the European oyster Ostrea edulis Linnaeus, 1758 and the introduced slipper limpet Crepidula fornicata Linnaeus, 1758 in the North Sea
Source: PLoS One. 2019 Oct 24;14(10):e0224249. doi: 10.1371/journal.pone.0224249 (PMC6812771; doi:10.1371/journal.pone.0224249)
Supplement: S6 Table — The test was calculated with the complete data set and with the oysters collected by Möbius removed. Provided are estimated coefficients, standard errors, t-values and p-values for collection years as a function of the number of shells collected. Note: high significance = ***; low significance = *; no significance = no asterisks. (DOCX) [file pone.0224249.s008.docx]

|  | Estimated coefficient | Standard error | t-value | p-value |
| --- | --- | --- | --- | --- |
| Complete data base | | | | |
| intercept | -32.21323 | 34.14796 | -0.943 | 0.347 |
| Year | 0.01958 | 0.01766 | 1.109 | 0.269 |
| Without Möbius oysters (1868 – 1885) | | | | |
| Intercept | -45.13829 | 52.59289 | -0.858 | 0.392 |
| Year | 0.02620 | 0.02704 | 0.969 | 0.334 |
